# Development and internal validation of a prediction model incorporating SOFA, Prognostic Nutritional Index, and Neutrophil-to-Albumin Ratio for early prediction of in-hospital mortality in patients with Sepsis: A single-center retrospective study

**DOI:** 10.1371/journal.pone.0353124

**Published:** 2026-07-30

**Authors:** Lutian Yi, Tao Yu, Xiaolu Zheng

**Affiliations:** 1 Department of Critical Care and Respiratory Medicine, The Affiliated People’s Hospital of Ningbo University, Ningbo, China; 2 Central Laboratory, The Affiliated People’s Hospital of Ningbo University, Ningbo, China; Children’s National Hospital, George Washington University, UNITED STATES OF AMERICA

## Abstract

**Objective:**

Sepsis prognosis is influenced by organ dysfunction, inflammation, and nutritional status. We aimed to develop and internally validate a prediction model incorporating the Sequential Organ Failure Assessment (SOFA) score, prognostic nutritional index (PNI), and neutrophil-to-albumin ratio (NAR) for early risk stratification of in-hospital mortality in patients with sepsis.

**Methods:**

We conducted a single-center retrospective study of 120 patients with sepsis. A multivariable logistic regression model was developed and internally validated using bootstrap resampling. Model performance was evaluated through discrimination, calibration, and decision curve analysis, with incremental predictive value assessed by net reclassification improvement and integrated discrimination improvement.

**Results:**

The SOFA score and NAR were independently associated with in-hospital mortality, while PNI did not reach statistical significance in multivariable analysis. The combined model demonstrated good discrimination with an area under the receiver operating characteristic curve of 0.904, higher than SOFA score alone (0.845) and quick SOFA (0.700), although the difference versus SOFA alone was not statistically significant (P = 0.075). Bootstrap internal validation yielded an optimism-corrected AUC of 0.870. Calibration was acceptable (Hosmer-Lemeshow P = 0.057; Brier score = 0.098). Decision curve analysis demonstrated greater net clinical benefit compared with SOFA score alone. The combined model showed potential improvement in risk reclassification (net reclassification improvement = 0.90; integrated discrimination improvement = 0.15; both P < 0.001), although its improvement in discrimination over the SOFA score alone did not reach statistical significance.

**Conclusions:**

The combined model showed statistically significant improvement in risk reclassification (net reclassification improvement = 0.90; integrated discrimination improvement = 0.15; both P < 0.001), although its improvement in overall discrimination (AUC) over the SOFA score alone did not reach statistical significance (P = 0.075).

## Introduction

Sepsis, defined as life-threatening organ dysfunction caused by a dysregulated host response to infection, remains a leading cause of morbidity and mortality in intensive care units (ICUs), particularly respiratory ICUs where pneumonia frequently precipitates both sepsis and respiratory failure [1,2]. Despite substantial advances in critical care management, outcomes remain poor due to the considerable clinical heterogeneity of sepsis, which impedes early risk stratification and personalized therapeutic interventions [3]. According to a 2017 global burden of disease study, sepsis accounted for approximately 48.9 million incident cases and 11.0 million deaths worldwide, representing nearly 20% of all global mortality [4]. Consequently, accurate early identification of high-risk patients upon hospital admission is essential for timely clinical escalation and optimal ICU resource allocation.

Although blood cultures remain the reference standard for identifying causative pathogens in sepsis, they suffer from long turnaround time, limited sensitivity, and difficulty in cultivating fastidious organisms [5,6]. Consequently, culture-independent biomarkers and clinical scoring systems have gained increasing attention for early risk stratification. Conventional markers including C-reactive protein (CRP) and procalcitonin (PCT) facilitate diagnosis but demonstrate limited prognostic utility [7]. Emerging evidence suggests that sepsis prognosis is determined not solely by the inciting pathogen but by the interplay between systemic inflammation and the patient’s physiological reserve. Hypoalbuminemia, prevalent in septic patients, correlates with adverse outcomes and reflects both malnutrition and the systemic inflammatory response [8].

These observations have prompted interest in composite biomarkers that capture multiple pathophysiological dimensions of sepsis. The prognostic nutritional index (PNI)—derived from serum albumin and absolute lymphocyte count—integrates nutritional status and immune competence [9,10]. Since albumin and lymphocyte measurements are routinely obtained at admission, PNI can be rapidly calculated without additional laboratory testing, making it practical for bedside risk assessment. The neutrophil-to-albumin ratio (NAR) similarly combines innate immune activation with nutritional reserve, offering a simple indicator of inflammatory burden relative to nutritional status [11,12]. The CRP/albumin ratio (CAR) has likewise demonstrated prognostic value in inflammatory and malignant conditions [13–15].

However, these inflammatory-nutritional markers do not directly quantify organ dysfunction, which constitutes a cardinal feature of the Sepsis-3 definition [1] and a principal determinant of prognosis. The Sequential Organ Failure Assessment (SOFA) score provides standardized quantification of organ dysfunction severity [1], and its prognostic utility in sepsis has been substantiated [16,17]. The quick SOFA (qSOFA), a simplified bedside version, remains widely adopted for early sepsis screening, although its predictive accuracy for in-hospital mortality varies considerably across cohorts. Recent reviews further support the clinical value of SOFA and related prognostic approaches in critical care [18].

A critical knowledge gap remains regarding whether integrated nutritional-inflammatory markers improve risk stratification beyond SOFA alone. Prior studies have characterized CAR and NAR as independent prognostic tools in sepsis [11–15], yet evidence supporting their combined use with SOFA remains limited. This issue is clinically relevant because patients with equivalent organ dysfunction severity frequently experience different outcomes, suggesting that nutritional reserve and inflammatory-immune balance may provide additional prognostic information. We therefore conducted a single-center retrospective cohort study to develop and internally validate a multivariable prediction model integrating SOFA, PNI, and NAR for early prediction of in-hospital mortality in patients with sepsis, and to assess its incremental prognostic value over SOFA alone.

## Materials and Methods

### Study design and setting

This single-center retrospective cohort study was conducted at a university-affiliated tertiary hospital. Patients were recruited from the intensive care unit (ICU), the respiratory intensive care unit (RICU), and the department of respiratory medicine. Consecutive adult patients admitted between January 2021 and December 2024 with a diagnosis of sepsis were screened for eligibility. The electronic medical records were accessed for research purposes on 25/3/2025.

### Participants

Patients were eligible if they met all of the following criteria: (1) age ≥ 18 years; (2) diagnosis of sepsis according to the Sepsis-3 clinical guidelines [1]; and (3) complete and retrievable electronic medical records. The diagnosis of sepsis was confirmed by experienced clinicians based on documented infection and sepsis-related organ dysfunction in accordance with Sepsis-3 criteria.

Patients were excluded if they met any of the following criteria: (1) pre-existing severe cardiovascular disease, including acute myocardial infarction or New York Heart Association class IV heart failure; or (2) concurrent immunosuppressive therapy or conditions likely to substantially confound outcomes, such as active malignancy, hematologic disorders, or human immunodeficiency virus infection.

### Ethical approval

This study was approved by the Institutional Ethics Committee of the hospital (Approval No. 2024 Research 029) and represents a secondary analysis of the approved retrospective cohort. Because of the retrospective observational design, the requirement for written informed consent was waived. All patient identifiers were removed before analysis, and the study was conducted in accordance with the Declaration of Helsinki.

### Data collection

Trained investigators extracted data from the electronic medical record system using a standardized data collection instrument. Baseline demographic characteristics, vital signs, infection sources, and comorbidities were systematically recorded. Laboratory parameters, including complete blood count with differential, serum albumin, C-reactive protein (CRP), procalcitonin, brain natriuretic peptide, and lactate, were measured within 24 h of admission, with preference given, when available, to values obtained before initiation of antimicrobial or vasopressor therapy.

The SOFA score was calculated using the worst values recorded during the initial 24 h after admission. The prognostic nutritional index (PNI) was calculated as serum albumin (g/L) + 5 × absolute lymphocyte count (×10⁹/L). The neutrophil-to-albumin ratio (NAR) was calculated as absolute neutrophil count (×10⁹/L)/ serum albumin (g/L). The CRP-to-albumin ratio (CAR) was calculated as serum CRP (mg/L)/ serum albumin (g/L). Complete clinical and laboratory data were available for all participants; therefore, no imputation was required for variables included in the final model.

### Outcome definition

The primary endpoint was in-hospital mortality, defined as death from any cause during the index hospitalization. Patients who died during hospitalization were classified as non-survivors, whereas those discharged alive with documented clinical improvement were classified as survivors. Patients transferred to other hospitals or discharged against medical advice were excluded from the analysis.

### Model development

Variable selection was based on clinical relevance and mechanistic plausibility. The SOFA score was retained as the reference measure of organ dysfunction severity. PNI and NAR were prespecified predictors because they reflect nutritional-immune homeostasis and inflammatory-nutritional balance, respectively, which are relevant to sepsis pathobiology but not directly captured by SOFA. Other markers, such as CAR, were evaluated descriptively and in univariate analyses but were not incorporated into the primary model.

Given the limited number of outcome events, a parsimonious multivariable logistic regression model was developed. Multicollinearity was assessed using the variance inflation factor (VIF); all values were <5, indicating no significant collinearity. Using the β coefficients from the multivariable logistic regression model (Table 2), the final prediction equation was constructed as follows: logit(p) = −1.842 + 0.581 × SOFA − 0.097 × PNI + 3.348 × NAR, where p denotes the predicted probability of in-hospital mortality. Predicted probability was calculated as p = 1/ (1 + e^ − logit(p)). Univariate logistic regression results for all candidate variables are presented in Supplementary Table 1.

### Model performance and internal validation

Discriminatory performance was assessed using the area under the receiver operating characteristic curve (AUC), with pairwise comparisons performed using the DeLong method. The combined model was compared with SOFA and quick Sequential Organ Failure Assessment (qSOFA) scores. Internal validation was performed using bootstrap resampling with 1,000 iterations. In each iteration, the model was refitted in a bootstrap sample and evaluated in the original cohort. Optimism was estimated as the difference between the mean bootstrap performance and the apparent performance, and an optimism-corrected AUC was then derived. Calibration was assessed using calibration plots, the Hosmer-Lemeshow goodness-of-fit test, and the Brier score. Decision curve analysis was performed to evaluate the clinical utility of the combined model relative to SOFA. Incremental predictive value was assessed using continuous net reclassification improvement (NRI) and integrated discrimination improvement (IDI). The 95% confidence intervals for NRI and IDI were not calculated; the reported P values were derived from 1,000 bootstrap resamples. The study was reported in accordance with the TRIPOD reporting guidelines [19].

### Statistical analysis

Statistical analyses were performed using SPSS version 25.0 (IBM Corp., Armonk, NY, USA) and R version 4.5.0 (R Foundation for Statistical Computing, Vienna, Austria). The following R packages were used: pROC for receiver operating characteristic analysis, rms for logistic regression modeling and bootstrap internal validation, rmda for decision curve analysis, and boot for bootstrap resampling. Continuous variables are expressed as mean ± standard deviation and compared using the independent samples t-test. Categorical variables are presented as counts and proportions and compared using the chi-square test. Optimal diagnostic thresholds were identified by maximizing the Youden index. A two-tailed P value <0.05 was considered statistically significant.

## Results

### Patient characteristics

The study cohort comprised 120 patients, with a mean age of 73.1 ± 12.7 years, including 55 men and 65 women. Stratification by in-hospital mortality yielded 80 survivors and 40 non-survivors. Baseline characteristics, including age, sex, vital signs, comorbidities, and infection sites, were generally comparable between groups; however, certain individual comorbidities and primary infection sources differed significantly (Table 1). Non-survivors had a higher admission respiratory rate and longer hospital stay than survivors.

**Table 1:** Comparison of clinical data between sepsis patients with good and poor in-hospital mortality.

Items	Poor prognosis groups (n = 40)	Good prognosis groups (n = 80)	t or X^2^	*P*
**Patient characteristics**				
Age (years)	74.20 ± 12.37	71.91 ± 13.64	0.923	0.359
Gender			0.193	0.661
Male [n(%)]	21 (52.5%)	44 (55.0%)		
Female [n(%)]	19 (47.5%)	36 (45.0%)		
Hospitalization days (day)	16.08 ± 8.89	11.68 ± 5.65	2.854	0.006
**Vital signs**				
SBP (mmHg)	106.85 ± 18.02	110.45 ± 16.27	−1.065	0.291
DBP (mmHg)	62.48 ± 10.68	65.84 ± 12.05	−1.556	0.123
MAP (mmHg)	77.26 ± 12.54	80.71 ± 12.15	−1.435	0.155
HR (bpm)	101.05 ± 8.63	99.15 ± 11.06	1.031	0.305
RR (bpm)	23.02 ± 3.35	21.65 ± 3.13	2.167	0.034
BT (°C)	37.38 ± 0.53	37.56 ± 0.53	−1.798	0.076
**Laboratory data**				
CRP (mg/L)	200.91 ± 100.71	160.12 ± 76.41	2.257	0.028
PCT (ng/mL)	32.93 ± 36.26	14.20 ± 24.58	2.946	0.005
Neutrophil count (×10⁹/L)	16.74 ± 7.19	11.62 ± 6.48	3.800	<0.001
BNP (pg/mL)	1346.72 ± 1466.48	344.78 ± 313.42	4.273	<0.001
Lac (mmol/L)	6.99 ± 5.20	2.38 ± 1.52	6.019	<0.001
SOFA Score	5.62 ± 3.33	2.51 ± 1.58	5.601	<0.001
qSOFA	1.35 ± 0.86	0.85 ± 0.70	3.182	0.002
CAR	7.83 ± 4.71	5.46 ± 2.84	2.915	0.005
NAR	0.65 ± 0.31	0.39 ± 0.23	4.503	<0.001
PNI	29.50 ± 5.68	34.30 ± 5.21	−4.486	<0.001
Lymphocyte count (×10⁹/L)	0.45 ± 0.35	0.85 ± 0.47	−5.213	<0.001
Albumin (g/L)	27.24 ± 5.44	30.05 ± 4.27	−2.857	0.006
**Underlying disease**				
Hypertension [n(%)]	24 (60.0%)	42 (52.5%)	—	0.560
Diabetes [n(%)]	11 (27.5%)	27 (33.8%)	—	0.538
Cardio-cerebrovascular diseases [n(%)]	8 (20.0%)	14 (17.5%)	—	0.804
Null (no underlying disease) [n(%)]	5 (12.5%)	13 (16.3%)	—	0.787
**Infection site**				
Respiratory system [n(%)]	20 (50.0%)	50 (62.5%)	—	0.239
Digestive system [n(%)]	3 (7.5%)	10 (12.5%)	—	0.540
Urinary system [n(%)]	3 (7.5%)	9 (11.3%)	—	0.749
Bloodstream infection [n(%)]	4 (10.0%)	7 (8.8%)	—	1.000
Soft tissue [n(%)]	6 (15.0%)	8 (10.0%)	—	0.547

Continuous variables are presented as mean ± SD and were compared using the independent-samples t-test. Categorical variables were compared using Fisher’s exact test when any expected cell count was < 5; otherwise, the chi-square test was applied. P values are two-tailed. A dash (—) in the test statistic column indicates that Fisher’s exact test was used, which does not produce a traditional test statistic.

### Laboratory findings and derived indices

Survivors and non-survivors differed significantly in inflammatory and metabolic parameters (Table 1). The non-survivor group had higher procalcitonin, CRP, neutrophil count, brain natriuretic peptide, and lactate levels, with lower lymphocyte count and serum albumin levels (all P < 0.01).

Regarding prognostic indices, non-survivors had a lower prognostic nutritional index (29.50 ± 5.68 vs. 34.30 ± 5.21, P < 0.001) and higher neutrophil-to-albumin ratio (0.65 ± 0.31 vs. 0.39 ± 0.23, P < 0.001) and CRP-to-albumin ratio (7.83 ± 4.71 vs. 5.46 ± 2.84, P < 0.001). Severity scores were also higher in non-survivors, with SOFA scores of 5.62 ± 3.33 versus 2.51 ± 1.58 (P < 0.001) and qSOFA scores of 1.35 ± 0.86 versus 0.85 ± 0.70 (P = 0.002).

### Independent predictors of in-hospital mortality

In multivariable logistic regression analysis, SOFA score (OR = 1.79 per point, 95% CI: 1.36–2.35, P < 0.001) and NAR (OR = 28.44 per unit, 95% CI: 3.03–267.17, P = 0.003) were independently associated with in-hospital mortality. PNI showed an inverse association with mortality but did not reach statistical significance (OR = 0.91 per unit, 95% CI: 0.80–1.03, P = 0.122). CAR was not retained in the final model after multivariable selection (Table 2).

**Table 2:** Multivariable logistic regression analysis for independent predictors of in-hospital mortality in patients with sepsis.

Variable	β coefficient	SE	Wald χ²	P Value	Adjusted OR	95% CI for OR
SOFA score	0.581	0.139	17.405	< 0.001	1.787	1.361–2.347
PNI	−0.097	0.062	2.393	0.122	0.908	0.803–1.026
NAR	3.348	1.143	8.584	0.003	28.444	3.028–267.166
Constant	−1.842	2.377	0.601	0.438	—	—

Abbreviations: SOFA, Sequential Organ Failure Assessment; PNI, prognostic nutritional index; NAR, neutrophil-to-albumin ratio; SE, standard error; OR, odds ratio; CI, confidence interval.

### Model Discrimination

**Fig 1:**
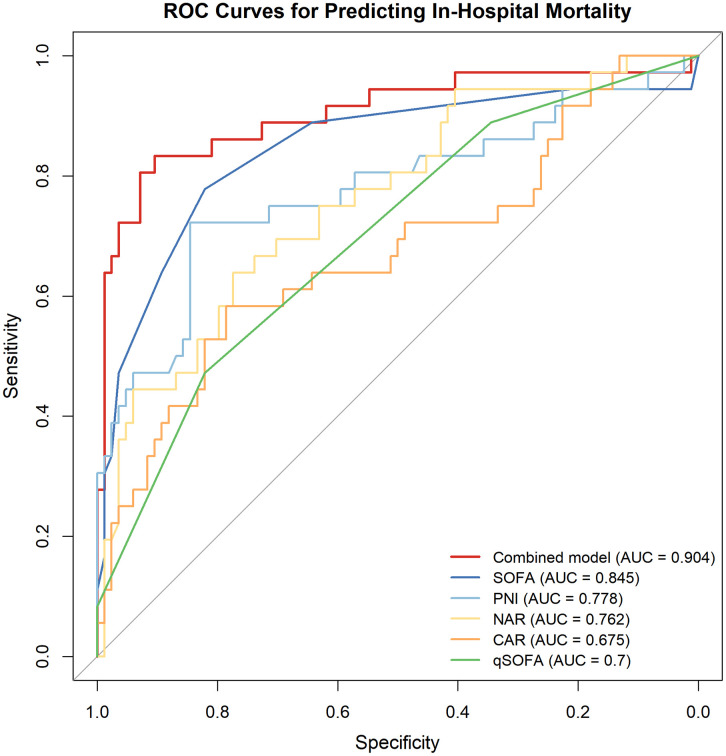
Receiver operating characteristic curves of the combined model (SOFA + PNI + NAR) and individual markers for predicting in-hospital mortality in patients with sepsis. The combined model showed greater discrimination than the individual markers. AUC, area under the curve; SOFA, Sequential Organ Failure Assessment; PNI, prognostic nutritional index; NAR, neutrophil-to-albumin ratio; CAR, C-reactive protein-to-albumin ratio. The numerical difference in AUC between the combined model and SOFA alone did not reach statistical significance (P = 0.075).

Receiver operating characteristic curve analysis was used to evaluate the discriminative performance of the combined model and its individual components (Fig 1, Table 3). The combined model (SOFA + PNI + NAR) achieved an area under the curve (AUC) of 0.904 (95% CI: 0.833–0.975). It showed better discrimination than qSOFA (AUC = 0.700; DeLong P < 0.001) and a numerically higher AUC than SOFA alone (AUC = 0.845; DeLong P = 0.075), although the latter difference was not statistically significant. Among the individual predictors, SOFA had the highest AUC (0.845), followed by PNI (0.778), NAR (0.762), and CAR (0.675). The optimal cutoff value for the combined model was 0.414, yielding a sensitivity of 83.3% and a specificity of 90.5%.

**Table 3:** Discriminatory performance of individual indicators and the combined model for predicting in-hospital mortality in patients with sepsis.

Indicator	AUC	95%CI	Cut-off value	Sensitivity (%)	Specificity (%)	Youden index
SOFA	0.845	0.757–0.933	≥3.5	77.8	82.1	0.599
PNI	0.778	0.675–0.881	<29.63	72.2	84.5	0.567
NAR	0.762	0.667–0.857	≥0.502	63.9	77.4	0.413
CAR	0.675	0.562–0.788	≥6.506	58.3	78.6	0.369
qSOFA	0.700	0.604–0.795	≥2	47.2	82.1	0.294
Combined model	0.904	0.833–0.975	≥0.414	83.3	90.5	0.738

Abbreviations: AUC, area under the receiver operating characteristic curve; CI, confidence interval; SOFA, Sequential Organ Failure Assessment; PNI, prognostic nutritional index; NAR, neutrophil-to-albumin ratio; CAR, C-reactive protein-to-albumin ratio; qSOFA, quick Sequential Organ Failure Assessment.

Notes: The cut-off value for the combined model refers to the optimal predicted probability threshold. The cut-off for qSOFA was set at ≥2 according to its original definition. These cut-off values were derived from the present sample and should be considered exploratory rather than definitive; they are not intended for clinical decision-making without external validation.

### Internal validation

Bootstrap internal validation using 1,000 resamples yielded an optimism-corrected AUC of 0.870, compared with an apparent AUC of 0.904 (optimism = 0.034; Table 3), indicating acceptable internal stability.

### Calibration

**Fig 2:**
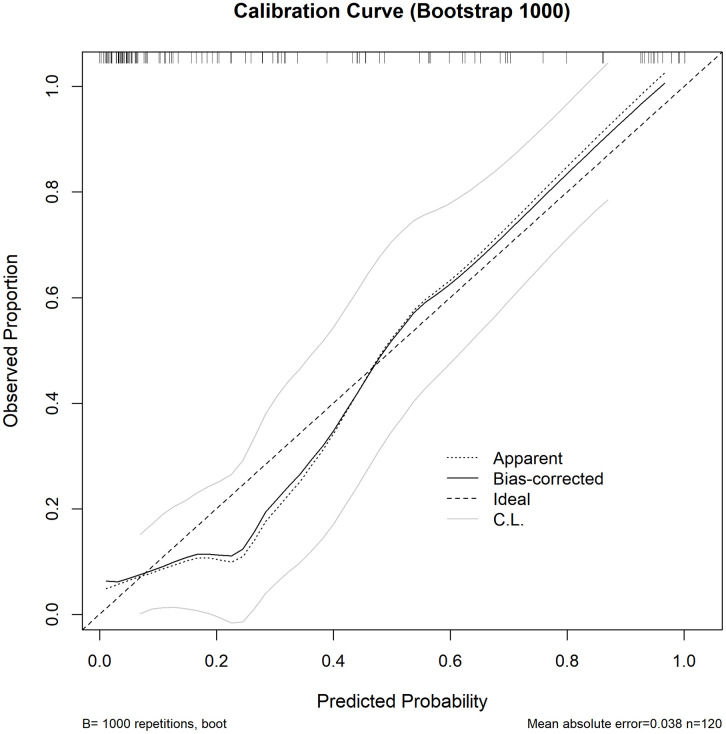
Calibration curve of the combined model (SOFA + PNI + NAR) for predicting in-hospital mortality in patients with sepsis. The plot was generated using 1,000 bootstrap resamples to assess agreement between predicted probabilities and observed outcomes. The 45° diagonal line indicates the ideal reference. The model curve showed reasonable agreement with the reference line across the range of predicted risk.

Calibration was evaluated using the Hosmer-Lemeshow goodness-of-fit test, bootstrap-corrected calibration metrics, calibration curves, and the Brier score. Bootstrap internal validation with 1,000 resamples yielded an optimism-corrected calibration slope of 0.92 and intercept of −0.04, indicating close agreement between predicted and observed mortality probabilities. The Hosmer-Lemeshow test showed no significant lack of fit (P = 0.057), the calibration curve demonstrated consistent agreement between predicted and observed outcomes across the entire range of risk (Fig 2), and the Brier score of 0.098 further confirmed favorable predictive accuracy.

### Clinical utility

**Fig 3:**
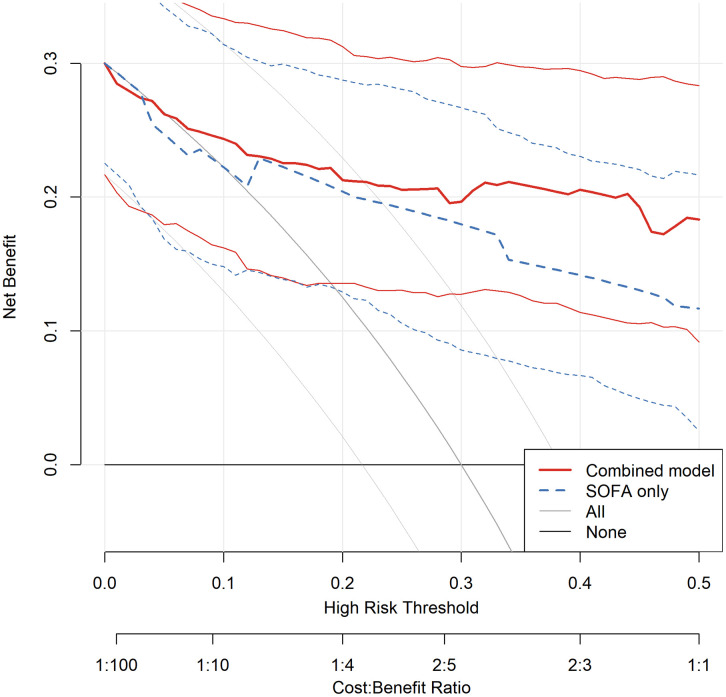
Decision curve analysis of the combined model (SOFA + PNI + NAR) versus SOFA alone for predicting in-hospital mortality in patients with sepsis. The y-axis represents net benefit, and the x-axis represents threshold probability. The combined model (red line) showed a higher net benefit than SOFA alone (blue line) across a clinically relevant range of threshold probabilities.

Decision curve analysis showed that the combined model provided greater net benefit than SOFA alone across a clinically relevant range of threshold probabilities for in-hospital mortality (Fig 3).

### Incremental predictive value

Compared with SOFA alone, the combined model showed improved risk reclassification. The continuous net reclassification improvement (NRI) was 0.90 (P < 0.001), and the integrated discrimination improvement (IDI) was 0.15 (P < 0.001).

### Survival analysis

**Fig 4:**
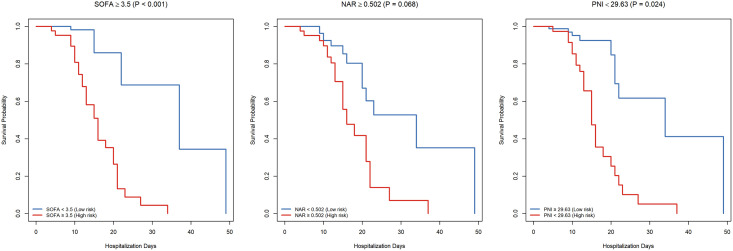
Kaplan–Meier curves of in-hospital survival in patients with sepsis, stratified by the optimal cut-off values of SOFA (≥ 3.5 vs. < 3.5), NAR (≥ 0.502 vs. < 0.502), and PNI (< 29.63 vs. ≥ 29.63). P values were calculated using the log-rank test.

Using ROC-derived optimal cut-off values (SOFA ≥ 3.5, NAR ≥ 0.502, and PNI < 29.63), Kaplan-Meier analysis demonstrated significant stratification of in-hospital mortality risk. Patients with SOFA ≥ 3.5 had lower survival probability than those with SOFA < 3.5 (log-rank P < 0.001). Patients with PNI < 29.63 also had lower in-hospital survival than those with PNI ≥ 29.63 (log-rank P = 0.024). The survival difference between patients with NAR ≥ 0.502 and those with NAR < 0.502 was not statistically significant (log-rank P = 0.068) (Fig 4).

## Discussion

This single-center retrospective study developed and internally validated a combined model integrating SOFA, PNI, and NAR to predict in-hospital mortality in septic patients. The model demonstrated acceptable discrimination and calibration with meaningful net benefit on decision curve analysis. Relative to SOFA alone, incorporation of PNI and NAR improved risk reclassification (NRI = 0.90, IDI = 0.15), suggesting that nutritional and inflammatory biomarkers contribute information beyond organ dysfunction scores.

The three components address distinct pathophysiological dimensions. SOFA quantifies organ dysfunction—a central determinant of sepsis prognosis. PNI integrates albumin and lymphocyte count, reflecting both nutritional reserve and adaptive immune capacity. NAR captures the ratio of systemic inflammation to nutritional status. By design, no single marker fully represents this complexity; their combination allowed the model to capture multifactorial risk that SOFA alone cannot.

SOFA is the reference standard for sepsis severity and consistently predicts mortality across populations [16,17]. In our cohort, SOFA was the single strongest predictor. Yet SOFA inherently lags: it reflects organ dysfunction that has already manifested clinically. The model’s additional components—PNI and NAR—operate on a different timescale, capturing nutritional depletion and inflammatory activation that may precede overt organ failure. Earlier detection of these processes could allow more timely intervention.

Prior work links PNI to sepsis outcomes through its dual reflection of nutritional and immune status [20–23]. Elevated NAR similarly associates with mortality [11,24]. In our multivariable analysis, PNI did not independently reach significance, yet its inclusion improved overall reclassification. NAR remained an independent predictor, though its wide confidence interval counsels caution. In a sensitivity analysis comparing the full model with a simplified two-variable model (SOFA + NAR), the full model showed only a minimal numerical increase in AUC (0.904 vs. 0.896), and neither NRI nor IDI indicated a statistically significant improvement (NRI = 0.17, P = 0.362; IDI = 0.02, P = 0.11). These results suggest that the incremental contribution of PNI beyond SOFA and NAR was limited in this cohort. Nevertheless, PNI was retained as a prespecified predictor because of its biological and clinical plausibility. The lack of statistical significance for NAR in univariable Kaplan–Meier analysis likely reflects loss of signal from dichotomization and small event count. CAR was excluded from the final model, a finding consistent with substantial overlap of its information with the three retained variables [13–15].

A key observation was that the combined model did not significantly exceed SOFA’s discrimination (DeLong P = 0.075). However, the NRI and IDI results—both statistically significant—indicate that risk reclassification occurred despite the marginal AUC difference. This dissociation reflects a real phenomenon: improvements in individual risk stratification need not always translate to large increments in overall discrimination. Conversely, when compared against qSOFA (a simpler bedside tool), our model performed substantially better (AUC 0.904 vs 0.700, P < 0.001), establishing that the complexity added meaningful discrimination relative to simplified screening scores. The plateau relative to SOFA likely reflects sample size constraints, the modest number of deaths, and potential redundancy among organ dysfunction, nutritional, and inflammatory measures in predicting this outcome.

We selected PNI over more complex nutritional indices such as mNUTRIC for practical reasons: it requires only baseline laboratory values routinely available at admission. Similarly, NAR demands no specialized assays. This design choice prioritized bedside applicability without sacrificing information gain.

Decision curve analysis reinforced the discriminative and reclassification findings: the combined model accrued net clinical benefit across the range of reasonable decision thresholds. Kaplan–Meier stratification was significant for SOFA and PNI independently, but not for NAR alone—an observation attributable to power limitations and the continuous nature of NAR in its native form. Collectively, these results position the combined model as a potential tool for earlier and more granular risk assessment in sepsis. The model may be applied at hospital admission to identify patients at elevated risk of in-hospital death who may warrant closer monitoring, although its role in guiding specific management decisions requires prospective validation. For bedside nurses in respiratory and critical care settings, this model may assist in early identification of high-risk septic patients, facilitating more intensive monitoring and timely communication with the medical team. Replication in external cohorts remains essential.

Several limitations warrant acknowledgment. First, the single-center design with modest sample size and outcome frequency constrains generalizability. With 40 in-hospital deaths and three prespecified predictors, the model development was broadly consistent with conventional EPV recommendations, although a larger number of events would still be desirable for more precise estimation. Internal validation mitigated but did not eliminate optimism bias. Second, external validation in independent cohorts is a mandatory next step. Third, the marginal statistical advantage over SOFA demands confirmation in larger samples before claims of superiority can be sustained. Fourth, a small subset of patients presented with SOFA scores of 0–1, reflecting real-world diagnostic heterogeneity at hospital entry. The exclusion criteria, while intended to reduce confounding, may limit the applicability of the model to sepsis patients with major comorbidities, who represent a substantial proportion of the real-world sepsis population. Fifth, comprehensive data for APACHE II or SAPS II were unavailable, precluding comparison with these alternative severity instruments. Sixth, the model was trained on baseline values only; dynamic biomarker trajectories may hold additional prognostic signal. PNI was retained in the model based on clinical plausibility and prior evidence linking nutritional status to sepsis outcomes, rather than on statistical significance alone. However, its independent association with in-hospital mortality was not confirmed in this cohort (P = 0.122). The wide confidence interval for NAR likely reflects the limited number of events, so this effect estimate should be interpreted cautiously. Finally, these findings remain exploratory and should not guide clinical decisions without prospective validation in multicenter cohorts.

## Conclusions

We evaluated SOFA, PNI, and NAR for predicting in-hospital mortality in septic patients. A combined model incorporating these three markers achieved acceptable discrimination (AUC 0.904) and demonstrated meaningful risk reclassification over SOFA alone (NRI = 0.90, IDI = 0.15). However, the gain in discrimination over SOFA alone was not statistically significant; nonetheless, the significant NRI and IDI indicate that PNI and NAR capture clinically relevant reclassification. Since all three variables are extractable from baseline admission data, the model should be easy to apply at admission. Single-center design and modest sample size limit generalizability; prospective multicenter validation is needed before clinical adoption.

## Supporting information

S1 Dataset Anonymized dataset for the prediction model. The dataset includes all variables used in the analysis (age, sex, vital signs, laboratory parameters, SOFA score, PNI, NAR, CAR, and in-hospital mortality outcome). Patient identifiers have been removed to protect privacy.
(CSV)
