# Supplementary material for: Development and internal validation of a prediction model incorporating SOFA, Prognostic Nutritional Index, and Neutrophil-to-Albumin Ratio for early prediction of in-hospital mortality in patients with Sepsis: A single-center retrospective study
Source: PLoS One. 2026 Jul 30;21(7):e0353124. doi: 10.1371/journal.pone.0353124 (PMC13422871; doi:10.1371/journal.pone.0353124)
Supplement: Attachment — Submitted filename: Response to Reviewers.docx [file pone.0353124.s003.docx]

Manuscript ID: PONE-D-26-25132

Title: Development and Internal Validation of a Prediction Model Incorporating SOFA, Prognostic Nutritional Index, and Neutrophil-to-Albumin Ratio for Early Prediction of In-Hospital Mortality in Patients with Sepsis: A Single-Center Retrospective Study

Corresponding Author: Lutian Yi

Dear Dr. Benjamin M. Liu and the Reviewers,

Thank you for your thoughtful and constructive comments on our manuscript. We have carefully addressed all the points raised by the Academic Editor and the reviewers. Below we provide a point-by-point response. All changes in the revised manuscript have been highlighted using track changes.

**Response to Academic Editor**

Comment:

***The authors should add introduction that blood cultures play a central diagnostic role in detecting sepsis, particularly when bloodstream infection (bacteremia or fungemia) is suspected but blood cultures suffer from long turnaround time, low sensitivity, requirement of specific culture conditions and difficulties of growing fastidious organisms. More references should be cited, with this two (PMID: 41246370; PMID: 34142857) as examples (citing is optional).***

Response:

We thank the Editor for this valuable suggestion. We agree that the limitations of blood cultures provide important context for the use of rapid, culture-independent biomarkers and clinical scoring systems. Accordingly, we have revised the Introduction by replacing the original opening sentence of the biomarker paragraph (“Biomarkers play a key role in assessing infection severity and prognosticating sepsis outcomes.”) with the following sentences (inserted immediately after the Sepsis-3 definition and before the discussion of conventional markers such as CRP and PCT):

“Although blood cultures remain the reference standard for identifying causative pathogens in sepsis, they suffer from long turnaround time, limited sensitivity, and difficulty in cultivating fastidious organisms [5,6]. Consequently, culture-independent biomarkers and clinical scoring systems have gained increasing attention for early risk stratification. Conventional markers including C-reactive protein (CRP) and procalcitonin (PCT) facilitate diagnosis but demonstrate limited prognostic utility [7]. Emerging evidence suggests that sepsis prognosis is determined not solely by the inciting pathogen but by the interplay between systemic inflammation and the patient’s physiological reserve. Hypoalbuminemia, prevalent in septic patients, correlates with adverse outcomes and reflects both malnutrition and the systemic inflammatory response [8].”

We have incorporated the two suggested references into the revised Introduction. All subsequent reference numbers have been updated throughout the manuscript accordingly. The revised text is highlighted in red in the tracked-changes version.

**Response to Reviewer #1**

Comment:

***The technique suggested by author can be accepted as a method for sepsis validation the data set is adequate. Such studies are a benefit to critical ill hospital patients. Very few articles available on above subject. The only problem I found with the manuscript is that the authors have not adequately explained or declared as to how they came up with the formula for the logistic regression (lines 131-132). I feel like this should be explained in the final manuscript.***

Response:

We thank the reviewer for this important comment. In the original manuscript, the logistic regression equation was presented without explicitly stating how it was derived. As shown in Table 2, the β coefficients for all model terms, including the intercept, were already reported. To make the derivation transparent, we have revised the Methods section (Model development subsection) in the marked-up manuscript, where the changes are highlighted in red. The revised text reads as follows:

“Given the limited number of outcome events, a parsimonious multivariable logistic regression model was developed. Multicollinearity was assessed using the variance inflation factor (VIF); all values were <5, indicating no significant collinearity. Using the β coefficients from the multivariable logistic regression model (Table 2), the final prediction equation was constructed as follows: logit(p) = −1.842 + 0.581 × SOFA − 0.097 × PNI + 3.348 × NAR, where p denotes the predicted probability of in-hospital mortality. Predicted probability was calculated as p = 1 / (1 + e^−logit(p)). Univariate logistic regression results for all candidate variables are presented in Supplementary Table 1.”

The coefficients in the equation are identical to those reported in Table 2. We believe this revision fully addresses the reviewer’s concern.

**Response to Reviewer #2**

Comment:

***The technique suggested by author can be accepted as a method for sepsis validation the data set is adequate. Such studies are a benefit to critical ill hospital patients. Very few articles available on above subject.***

Response:

We sincerely thank the reviewer for the positive and encouraging comments. We are pleased that the reviewer found our study and dataset appropriate for this purpose and recognized the potential clinical value of the model in critically ill patients with sepsis. We agree that further external validation will be important before routine clinical application, and we hope our work may serve as a useful foundation for future studies in this area.

**Response to Journal Requirements**

1. ***PLOS ONE style requirements and file naming***

We have checked the PLOS ONE style requirements and file naming conventions and confirm that the revised files have been prepared accordingly.

2. ***Code sharing guidelines***

We have reviewed *PLOS ONE*’s code sharing policy. No author-generated custom code was developed for this study. All statistical analyses were performed using standard functions in SPSS version 25.0 (IBM Corp.) and R version 4.5.0 with publicly available packages (pROC, rms, rmda, boot). Therefore, code sharing does not apply to this submission.

3. ***Funding statement removal from Acknowledgments***

We have removed all funding-related text from the manuscript. The Acknowledgments section now contains only non-financial acknowledgments and does not include any funding information. Funding information appears only in the Funding Statement in the online submission system, as required.

4. ***Matching grant information***

We confirm that the correct grant number is 2024KY369 from the Zhejiang Provincial Medical and Health Research Program. The same grant information has been provided consistently in the submission materials.

5. ***Role of funder***

The funder had no role in study design, data collection and analysis, decision to publish, or preparation of the manuscript. This statement has been included in the cover letter as requested.

6. ***Data availability statement***

The anonymized dataset used for this analysis has been provided as Supporting Information (S1 Dataset). No third-party contact is required.

7. ***ORCID iD for corresponding author***

The corresponding author has created and validated an ORCID iD in Editorial Manager via the “Update my Information → Fetch/Validate” link.

8. ***Ethics statement location***

We confirm that the ethics statement appears only in the Methods section under the subheading “Ethical Approval.” The duplicate ethics statement previously located in the Declarations section has been removed.

9. ***Citation of recommended works***

We have cited the two references recommended by the Academic Editor (PMID: 41246370 and PMID: 34142857) in the revised Introduction (now numbered as [5] and [6]). These references are relevant to the added discussion on blood culture limitations. No other reviewer-recommended citations were requested.

10. ***Reference list completeness and retractions***

We have carefully reviewed the reference list and made the necessary corrections to ensure completeness and accuracy. We also verified that none of the cited references have been retracted.

We believe the revised manuscript is now suitable for publication in PLOS ONE. We thank the Academic Editor and reviewers again for their time and valuable input.

Sincerely,

Lutian Yi, MD

Corresponding author
