# Supplementary material for: Development and internal validation of a prediction model incorporating SOFA, Prognostic Nutritional Index, and Neutrophil-to-Albumin Ratio for early prediction of in-hospital mortality in patients with Sepsis: A single-center retrospective study
Source: PLoS One. 2026 Jul 30;21(7):e0353124. doi: 10.1371/journal.pone.0353124 (PMC13422871; doi:10.1371/journal.pone.0353124)
Supplement: Peer review history

# Peer review: Decision Letter 0

**DOI:** 10.1371/journal.pone.0353124.r001

**Authors:** Benjamin Liu

14 Jun 2026

PONE-D-26-25132

Development and Internal Validation of a Prediction Model Incorporating SOFA, Prognostic Nutritional Index, and Neutrophil-to-Albumin Ratio for Early Prediction of In-Hospital Mortality in Patients with Sepsis: A Single-Center Retrospective Study

PLOS One

Dear Dr. Yi,

Thank you for submitting your manuscript to PLOS ONE. After careful consideration, we feel that it has merit but does not fully meet PLOS ONE’s publication criteria as it currently stands. Therefore, we invite you to submit a revised version of the manuscript that addresses the points raised during the review process.

**ACADEMIC EDITOR'S COMMENTS:**

The authors should add introduction that blood cultures play a central diagnostic role in detecting sepsis, particularly when bloodstream infection (bacteremia or fungemia) is suspected but blood cultures suffer from long turnaround time, low sensitivity, requirement of specific culture conditions and difficulties of growing fastidious organisms. More references should be cited, with this two (PMID: 41246370; PMID: 34142857) as examples (citing is optional).

Please submit your revised manuscript by Jul 14 2026 11:59PM. If you will need more time than this to complete your revisions, please reply to this message or contact the journal office at plosone@plos.org. When you're ready to submit your revision, log on to https://www.editorialmanager.com/pone/ and select the 'Submissions Needing Revision' folder to locate your manuscript file.

Please include the following items when submitting your revised manuscript:

- A letter that responds to each point raised by the academic editor and reviewer(s). You should upload this letter as a separate file labeled 'Response to Reviewers'.
- A marked-up copy of your manuscript that highlights changes made to the original version. You should upload this as a separate file labeled 'Revised Manuscript with Track Changes'.
- An unmarked version of your revised paper without tracked changes. You should upload this as a separate file labeled 'Manuscript'.

If you would like to make changes to your financial disclosure, please include your updated statement in your cover letter. Guidelines for resubmitting your figure files are available below the reviewer comments at the end of this letter.

If applicable, we recommend that you deposit your laboratory protocols in protocols.io to enhance the reproducibility of your results. Protocols.io assigns your protocol its own identifier (DOI) so that it can be cited independently in the future. For instructions see: https://journals.plos.org/plosone/s/submission-guidelines#loc-laboratory-protocols. Additionally, PLOS ONE offers an option for publishing peer-reviewed Lab Protocol articles, which describe protocols hosted on protocols.io. Read more information on sharing protocols at https://plos.org/protocols?utm_medium=editorial-email&utm_source=authorletters&utm_campaign=protocols.

As the corresponding author, your ORCID iD is verified in the submission system and will appear in the published article. PLOS supports the use of ORCID, and we encourage all coauthors to register for an ORCID iD and use it as well. Please encourage your coauthors to verify their ORCID iD within the submission system before final acceptance, as unverified ORCID iDs will not appear in the published article. *Only*  the individual author can complete the verification step; PLOS staff *cannot*  verify ORCID iDs on behalf of authors.

We look forward to receiving your revised manuscript.

Kind regards,

Benjamin M. Liu, PhD, D(ABMM), MB(ASCP)

Academic Editor

PLOS One

Journal Requirements:

When submitting your revision, we need you to address these additional requirements.

1. Please ensure that your manuscript meets PLOS ONE's style requirements, including those for file naming. The PLOS ONE style templates can be found at

https://journals.plos.org/plosone/s/file?id=wjVg/PLOSOne_formatting_sample_main_body.pdf and

https://journals.plos.org/plosone/s/file?id=ba62/PLOSOne_formatting_sample_title_authors_affiliations.pdf

2. Please note that PLOS One has specific guidelines on code sharing for submissions in which author-generated code underpins the findings in the manuscript. In these cases, we expect all author-generated code to be made available without restrictions upon publication of the work. Please review our guidelines at https://journals.plos.org/plosone/s/materials-and-software-sharing#loc-sharing-code and ensure that your code is shared in a way that follows best practice and facilitates reproducibility and reuse.

3. Thank you for stating the following in the Acknowledgments Section of your manuscript:

“This work was supported by the Zhejiang Provincial Medical and Health Research Program (Grant No. 2024KY369). The funder had no role in study design, data collection and analysis, decision to publish, or preparation of the manuscript.”

We note that you have provided funding information that is not currently declared in your Funding Statement. However, funding information should not appear in the Acknowledgments section or other areas of your manuscript. We will only publish funding information present in the Funding Statement section of the online submission form.

Please remove any funding-related text from the manuscript and let us know how you would like to update your Funding Statement. Currently, your Funding Statement reads as follows:

“This work was supported by the Zhejiang Provincial Medical and Health Research Program (Grant No. 2024KY369).”

Please include your amended statements within your cover letter; we will change the online submission form on your behalf.

4. We note that the grant information you provided in the ‘Funding Information’ and ‘Financial Disclosure’ sections do not match.

When you resubmit, please ensure that you provide the correct grant numbers for the awards you received for your study in the ‘Funding Information’ section.

5. Thank you for stating the following financial disclosure:

“This work was supported by the Zhejiang Provincial Medical and Health Research Program (Grant No. 2024KY369).”

Please state what role the funders took in the study.  If the funders had no role, please state: "The funders had no role in study design, data collection and analysis, decision to publish, or preparation of the manuscript."

If this statement is not correct you must amend it as needed.

Please include this amended Role of Funder statement in your cover letter; we will change the online submission form on your behalf.

6. In the online submission form you indicate that your data is not available for proprietary reasons and have provided a contact point for accessing this data. Please note that your current contact point is a co-author on this manuscript. According to our Data Policy, the contact point must not be an author on the manuscript and must be an institutional contact, ideally not an individual. Please revise your data statement to a non-author institutional point of contact, such as a data access or ethics committee, and send this to us via return email. Please also include contact information for the third party organization, and please include the full citation of where the data can be found.

7. PLOS requires an ORCID iD for the corresponding author in Editorial Manager on papers submitted after December 6th, 2016. Please ensure that you have an ORCID iD and that it is validated in Editorial Manager. To do this, go to ‘Update my Information’ (in the upper left-hand corner of the main menu), and click on the Fetch/Validate link next to the ORCID field. This will take you to the ORCID site and allow you to create a new iD or authenticate a pre-existing iD in Editorial Manager.

8. Your ethics statement should only appear in the Methods section of your manuscript. If your ethics statement is written in any section besides the Methods, please move it to the Methods section and delete it from any other section. Please ensure that your ethics statement is included in your manuscript, as the ethics statement entered into the online submission form will not be published alongside your manuscript.

9. If the reviewer comments include a recommendation to cite specific previously published works, please review and evaluate these publications to determine whether they are relevant and should be cited. There is no requirement to cite these works unless the editor has indicated otherwise.

10. Please review your reference list to ensure that it is complete and correct. If you have cited papers that have been retracted, please include the rationale for doing so in the manuscript text, or remove these references and replace them with relevant current references. Any changes to the reference list should be mentioned in the rebuttal letter that accompanies your revised manuscript. If you need to cite a retracted article, indicate the article’s retracted status in the References list and also include a citation and full reference for the retraction notice.

[Note: HTML markup is below. Please do not edit.]

Reviewers' comments:

Reviewer's Responses to Questions

**Comments to the Author**

1. Is the manuscript technically sound, and do the data support the conclusions?

The manuscript must describe a technically sound piece of scientific research with data that supports the conclusions. Experiments must have been conducted rigorously, with appropriate controls, replication, and sample sizes. The conclusions must be drawn appropriately based on the data presented.

Reviewer #1: Yes

Reviewer #2: Yes

**********

2. Has the statistical analysis been performed appropriately and rigorously?

Reviewer #1: Yes

Reviewer #2: Yes

**********

3. Have the authors made all data underlying the findings in their manuscript fully available?

The PLOS Data policy requires authors to make all data underlying the findings described in their manuscript fully available without restriction, with rare exception (please refer to the Data Availability Statement in the manuscript PDF file). The data should be provided as part of the manuscript or its supporting information, or deposited to a public repository. For example, in addition to summary statistics, the data points behind means, medians and variance measures should be available. If there are restrictions on publicly sharing data—e.g. participant privacy or use of data from a third party—those must be specified.

Reviewer #1: Yes

Reviewer #2: Yes

**********

4. Is the manuscript presented in an intelligible fashion and written in standard English?

PLOS ONE does not copyedit accepted manuscripts, so the language in submitted articles must be clear, correct, and unambiguous. Any typographical or grammatical errors should be corrected at revision, so please note any specific errors here.

Reviewer #1: Yes

Reviewer #2: Yes

**********

5. Review Comments to the Author

Please use the space provided to explain your answers to the questions above. You may also include additional comments for the author, including concerns about dual publication, research ethics, or publication ethics. (Please upload your review as an attachment if it exceeds 20,000 characters)

Reviewer #1: The manuscript is scientifically and technically sound and well written. The statistical analysis has been adequately performed and the statistics are presented in a simple fashion. The only problem I found with the manuscript is that the authors have not adequately explained or declared as to how they came up with the formula for the logistic regression (lines 131-132). I feel like this should be explained in the final manuscript.

Reviewer #2: The technique suggested by author can be accepted as a method for sepsis validation the data set is adequate. Such studies are a benefit to critical I'll hospital patients. Very few articles available on above subject.

**********

6. PLOS authors have the option to publish the peer review history of their article (what does this mean?). If published, this will include your full peer review and any attached files.

If you choose “no”, your identity will remain anonymous but your review may still be made public.

**Do you want your identity to be public for this peer review?**  For information about this choice, including consent withdrawal, please see our Privacy Policy.

Reviewer #1: No

Reviewer #2: **Yes:** Jayant balani

**********

[NOTE: If reviewer comments were submitted as an attachment file, they will be attached to this email and accessible via the submission site. Please log into your account, locate the manuscript record, and check for the action link "View Attachments". If this link does not appear, there are no attachment files.]

To ensure your figures meet our technical requirements, please review our figure guidelines: https://journals.plos.org/plosone/s/figures

You may also use PLOS’s free figure tool, NAAS, to help you prepare publication quality figures: https://journals.plos.org/plosone/s/figures#loc-tools-for-figure-preparation.

NAAS will assess whether your figures meet our technical requirements by comparing each figure against our figure specifications.

# Author comment: Author response to Decision Letter 1

**DOI:** 10.1371/journal.pone.0353124.r002

16 Jun 2026

Manuscript ID: PONE-D-26-25132

Title: Development and Internal Validation of a Prediction Model Incorporating SOFA, Prognostic Nutritional Index, and Neutrophil-to-Albumin Ratio for Early Prediction of In-Hospital Mortality in Patients with Sepsis: A Single-Center Retrospective Study

Corresponding Author: Lutian Yi

Dear Dr. Benjamin M. Liu and the Reviewers,

Thank you for your thoughtful and constructive comments on our manuscript. We have carefully addressed all the points raised by the Academic Editor and the reviewers. Below we provide a point-by-point response. All changes in the revised manuscript have been highlighted using track changes.

Response to Academic Editor

Comment:

The authors should add introduction that blood cultures play a central diagnostic role in detecting sepsis, particularly when bloodstream infection (bacteremia or fungemia) is suspected but blood cultures suffer from long turnaround time, low sensitivity, requirement of specific culture conditions and difficulties of growing fastidious organisms. More references should be cited, with this two (PMID: 41246370; PMID: 34142857) as examples (citing is optional).

Response:

We thank the Editor for this valuable suggestion. We agree that the limitations of blood cultures provide important context for the use of rapid, culture-independent biomarkers and clinical scoring systems. Accordingly, we have revised the Introduction by replacing the original opening sentence of the biomarker paragraph (“Biomarkers play a key role in assessing infection severity and prognosticating sepsis outcomes.”) with the following sentences (inserted immediately after the Sepsis-3 definition and before the discussion of conventional markers such as CRP and PCT):

“Although blood cultures remain the reference standard for identifying causative pathogens in sepsis, they suffer from long turnaround time, limited sensitivity, and difficulty in cultivating fastidious organisms [5,6]. Consequently, culture-independent biomarkers and clinical scoring systems have gained increasing attention for early risk stratification. Conventional markers including C-reactive protein (CRP) and procalcitonin (PCT) facilitate diagnosis but demonstrate limited prognostic utility [7]. Emerging evidence suggests that sepsis prognosis is determined not solely by the inciting pathogen but by the interplay between systemic inflammation and the patient’s physiological reserve. Hypoalbuminemia, prevalent in septic patients, correlates with adverse outcomes and reflects both malnutrition and the systemic inflammatory response [8].”

We have incorporated the two suggested references into the revised Introduction. All subsequent reference numbers have been updated throughout the manuscript accordingly. The revised text is highlighted in red in the tracked-changes version.

Response to Reviewer #1

Comment:

The technique suggested by author can be accepted as a method for sepsis validation the data set is adequate. Such studies are a benefit to critical ill hospital patients. Very few articles available on above subject. The only problem I found with the manuscript is that the authors have not adequately explained or declared as to how they came up with the formula for the logistic regression (lines 131-132). I feel like this should be explained in the final manuscript.

Response:

We thank the reviewer for this important comment. In the original manuscript, the logistic regression equation was presented without explicitly stating how it was derived. As shown in Table 2, the β coefficients for all model terms, including the intercept, were already reported. To make the derivation transparent, we have revised the Methods section (Model development subsection) in the marked-up manuscript, where the changes are highlighted in red. The revised text reads as follows:

“Given the limited number of outcome events, a parsimonious multivariable logistic regression model was developed. Multicollinearity was assessed using the variance inflation factor (VIF); all values were <5, indicating no significant collinearity. Using the β coefficients from the multivariable logistic regression model (Table 2), the final prediction equation was constructed as follows: logit(p) = −1.842 + 0.581 × SOFA − 0.097 × PNI + 3.348 × NAR, where p denotes the predicted probability of in-hospital mortality. Predicted probability was calculated as p = 1 / (1 + e^−logit(p)). Univariate logistic regression results for all candidate variables are presented in Supplementary Table 1.”

The coefficients in the equation are identical to those reported in Table 2. We believe this revision fully addresses the reviewer’s concern.

Response to Reviewer #2

Comment:

The technique suggested by author can be accepted as a method for sepsis validation the data set is adequate. Such studies are a benefit to critical ill hospital patients. Very few articles available on above subject.

Response:

We sincerely thank the reviewer for the positive and encouraging comments. We are pleased that the reviewer found our study and dataset appropriate for this purpose and recognized the potential clinical value of the model in critically ill patients with sepsis. We agree that further external validation will be important before routine clinical application, and we hope our work may serve as a useful foundation for future studies in this area.

Response to Journal Requirements

1.PLOS ONE style requirements and file naming

We have checked the PLOS ONE style requirements and file naming conventions and confirm that the revised files have been prepared accordingly.

2. Code sharing guidelines

We have reviewed PLOS ONE’s code sharing policy. No author-generated custom code was developed for this study. All statistical analyses were performed using standard functions in SPSS version 25.0 (IBM Corp.) and R version 4.5.0 with publicly available packages (pROC, rms, rmda, boot). Therefore, code sharing does not apply to this submission.

3. Funding statement removal from Acknowledgments

We have removed all funding-related text from the manuscript. The Acknowledgments section now contains only non-financial acknowledgments and does not include any funding information. Funding information appears only in the Funding Statement in the online submission system, as required.

4. Matching grant information

We confirm that the correct grant number is 2024KY369 from the Zhejiang Provincial Medical and Health Research Program. The same grant information has been provided consistently in the submission materials.

5. Role of funder

The funder had no role in study design, data collection and analysis, decision to publish, or preparation of the manuscript. This statement has been included in the cover letter as requested.

6. Data availability statement

The anonymized dataset used for this analysis has been provided as Supporting Information (S1 Dataset). No third-party contact is required.

7. ORCID iD for corresponding author

The corresponding author has created and validated an ORCID iD in Editorial Manager via the “Update my Information → Fetch/Validate” link.

8. Ethics statement location

We confirm that the ethics statement appears only in the Methods section under the subheading “Ethical Approval.” The duplicate ethics statement previously located in the Declarations section has been removed.

9. Citation of recommended works

We have cited the two references recommended by the Academic Editor (PMID: 41246370 and PMID: 34142857) in the revised Introduction (now numbered as [5] and [6]). These references are relevant to the added discussion on blood culture limitations. No other reviewer-recommended citations were requested.

10. Reference list completeness and retractions

We have carefully reviewed the reference list and made the necessary corrections to ensure completeness and accuracy. We also verified that none of the cited references have been retracted.

We believe the revised manuscript is now suitable for publication in PLOS ONE. We thank the Academic Editor and reviewers again for their time and valuable input.

Sincerely,

Lutian Yi, MD

Corresponding author

Attachment Submitted filename: Response to Reviewers.docx

# Editor report: Decision Letter 1

**DOI:** 10.1371/journal.pone.0353124.r003

**Authors:** Benjamin Liu

18 Jun 2026

Development and Internal Validation of a Prediction Model Incorporating SOFA, Prognostic Nutritional Index, and Neutrophil-to-Albumin Ratio for Early Prediction of In-Hospital Mortality in Patients with Sepsis: A Single-Center Retrospective Study

PONE-D-26-25132R1

Dear Dr. Yi,

We’re pleased to inform you that your manuscript has been judged scientifically suitable for publication and will be formally accepted for publication once it meets all outstanding technical requirements.

Within one week, you’ll receive an e-mail detailing the required amendments. When these have been addressed, you’ll receive a formal acceptance letter and your manuscript will be scheduled for publication.

An invoice will be generated when your article is formally accepted. Please note, if your institution has a publishing partnership with PLOS and your article meets the relevant criteria, all or part of your publication costs will be covered. Please make sure your user information is up-to-date by logging into Editorial Manager at Editorial Manager® and clicking the ‘Update My Information' link at the top of the page. For questions related to billing, please contact billing support.

If your institution or institutions have a press office, please notify them about your upcoming paper to help maximize its impact. If they’ll be preparing press materials, please inform our press team as soon as possible -- no later than 48 hours after receiving the formal acceptance. Your manuscript will remain under strict press embargo until 2 pm Eastern Time on the date of publication. For more information, please contact onepress@plos.org.

Kind regards,

Benjamin M. Liu, PhD, D(ABMM), MB(ASCP)

Academic Editor

PLOS One

Additional Editor Comments (optional):

Reviewers' comments:

# Editor report: Acceptance letter

**DOI:** 10.1371/journal.pone.0353124.r004

**Authors:** Benjamin Liu

PONE-D-26-25132R1

PLOS One

Dear Dr. Yi,

I'm pleased to inform you that your manuscript has been deemed suitable for publication in PLOS One. Congratulations! Your manuscript is now being handed over to our production team.

At this stage, our production department will prepare your paper for publication. This includes ensuring the following:

* All references, tables, and figures are properly cited

* All relevant supporting information is included in the manuscript submission,

* There are no issues that prevent the paper from being properly typeset

You will receive further instructions from the production team, including instructions on how to review your proof when it is ready. Please keep in mind that we are working through a large volume of accepted articles, so please give us a few days to review your paper and let you know the next and final steps.

Lastly, if your institution or institutions have a press office, please let them know about your upcoming paper now to help maximize its impact. If they'll be preparing press materials, please inform our press team within the next 48 hours. Your manuscript will remain under strict press embargo until 2 pm Eastern Time on the date of publication. For more information, please contact onepress@plos.org.

You will receive an invoice from PLOS for your publication fee after your manuscript has reached the completed accept phase. If you receive an email requesting payment before acceptance or for any other service, this may be a phishing scheme. Learn how to identify phishing emails and protect your accounts at https://explore.plos.org/phishing.

If we can help with anything else, please email us at customercare@plos.org.

Thank you for submitting your work to PLOS ONE and supporting open access.

Kind regards,

PLOS ONE Editorial Office Staff

on behalf of

Dr. Benjamin M. Liu

Academic Editor

PLOS One
